# Hybrid operating rooms and the risk of postoperative hypothermia in pregnant women with placenta previa: A retrospective cohort study

**DOI:** 10.1371/journal.pone.0305951

**Published:** 2024-06-25

**Authors:** Sou Hyun Lee, You Hyun Lee

**Affiliations:** 1 Department of Anesthesiology and Pain Medicine, Kyungpook National University Hospital, Daegu, Republic of Korea; 2 Department of Ophthalmology, Daegu Dongsan Medical Center, Keimyung University School of Medicine, Daegu, Republic of Korea; IRCCS: IRCCS Ospedale San Raffaele, ITALY

## Abstract

**Background:**

Births at advanced maternal ages (≥ 35 years) are increasing. This has been associated with a higher incidence of placenta previa, which increases bleeding risk. Hybrid operating rooms, designed to accommodate interventions and cesarean sections, are becoming more prominent because of their dual capabilities and benefits. However, they have been associated with increased postoperative hypothermia in pediatric settings; moreover, this has not been studied in pregnant women with placenta previa.

**Methods:**

This retrospective cohort study included pregnant women diagnosed with placenta previa who underwent elective cesarean section under general anesthesia between May 2019 and 2023. The patients were categorized according to the operating room type. The primary outcome was to determine whether the hybrid operating room is a risk factor for immediate postoperative hypothermia, defined as a tympanic membrane temperature below 36.0°C. The secondary outcomes were the effects of immediate postoperative hypothermia on the durations of postanesthetic care unit and postoperative hospital stays and incidence of complications.

**Results:**

Immediate postoperative hypothermia (tympanic membrane temperature < 36.0°C) was more prevalent in the hybrid than in the standard operating room group (20% vs. 36.6%, p = 0.033), with a relative risk of 2.86 (95% confidence interval 1.24–6.64, p < 0.001). Patients undergoing surgery in the hybrid operating room who experienced immediate postoperative hypothermia stayed longer in the postanesthetic care unit (26 min vs. 40 min, p < 0.001) and in the hospital after surgery (4 days; range 3–5 vs. 4 days; range 4–11, p = 0.021). However, the complication rates of both groups were not significantly different (11.3% vs 7.3%, p = 0.743).

**Conclusion:**

Hybrid operating rooms may increase the risk of postoperative hypothermia. Postoperative hypothermia is associated with prolonged postanesthetic care unit and hospital stays. Preventing hypothermia in patients in hybrid operating rooms is of utmost importance.

## Introduction

A noticeable increase has been observed in births among women of advanced maternal age (≥ 35 years) [1, 2]. In South Korea, 35.7% of pregnancies were among women in this age group in 2022 [3]. This increase in births at advanced maternal age is associated with the increased incidence of placenta previa [4], which is associated with an increased risk of bleeding [5]. To address this, the bilateral internal iliac artery balloon technique was introduced in 2007, and it is specifically designed to mitigate bleeding during cesarean sections [6]. Consequently, hybrid operating rooms (OR) equipped for interventions and cesarean sections are important [7]. In addition, performing procedures and surgeries simultaneously in a hybrid OR has the added benefit of reduced radiation exposure compared with performing procedures in a remote intervention room or surgery in a standard OR, which is beneficial for the fetus [8].

Recent studies focusing on pediatric patients have revealed a high incidence of postoperative hypothermia in hybrid ORs [9, 10]. Postoperative hypothermia is associated with extended stays in the postanesthetic care unit and prolonged hospitalization [11–13]. Given the higher installation and operational costs of hybrid ORs than those of standard ORs [14] and the expanding market for hybrid ORs [15], an increase in the number of procedures and surgeries for pregnant patients diagnosed with placenta previa in hybrid ORs is anticipated. Therefore, it is important to investigate whether the combined interventions and treatments in hybrid ORs increase the risk of postoperative hypothermia in pregnant women with placenta previa. To the best of our knowledge, no studies have been conducted on this topic.

This study aimed to determine whether the choice of OR influences the incidence of postoperative hypothermia. We hypothesized that hybrid ORs are associated with postoperative hypothermia.

## Materials and methods

This retrospective cohort study was approved by the Institutional Review Board of Keimyung University Dongsan Medical Center (approval number: 2023-07-050; July 31, 2023). The requirement for patient consent was waived because the study was retrospective. Data access for this study was conducted on August 15, 2023.

### Participants

The patient cohort for the study was identified from the Clinical Data Warehouse version 2.5 (CDW, Planit Healthcare, Seoul, Korea), and the data were sourced directly from electronic medical records. This manuscript adhered to the Strengthening the Reporting of Observational Studies in Epidemiology (STROBE) guidelines.

Pregnant individuals diagnosed with one of the International Classification of Diseases Tenth Revision (ICD-10) codes were eligible for inclusion in this study: O44, O440, or O441 (placenta previa, placenta previa specified as without hemorrhage, placenta previa with hemorrhage). The included patients underwent bilateral internal iliac artery balloon occlusion on the day of surgery and elective cesarean section under general anesthesia between May 2019 and May 2023. Patients who underwent bilateral internal iliac artery balloon occlusion after surgery were excluded.

The patients were categorized into two groups. The hybrid OR group comprised patients who underwent simultaneous bilateral internal iliac artery balloon occlusion using C-arm guidance, followed by a cesarean section in a hybrid OR. The standard OR group comprised patients who first underwent bilateral internal iliac artery balloon occlusion in a remote intervention room before undergoing surgery in a standard OR.

### Standard anesthetic procedure for cesarean section in patients with placenta previa

The hybrid and standard ORs were maintained at a room temperature of 25°C, with a humidity of 50–60%. For patients undergoing cesarean section in the hybrid OR, awake 20-G right radial arterial line and 7Fr three lumen right internal jugular central line catheterizations were performed using a linear ultrasound probe. Internal iliac artery ballooning procedures were performed by an interventional radiologist, and the detailed procedure has been previously described [16]. After the ballooning procedure and immediately before surgery, propofol (2 mg/kg) and succinylcholine (1.5 mg/kg) were administered. After 30–60 s, endotracheal intubation was performed, followed by the administration of rocuronium (0.6 mg/kg). Anesthesia was maintained using a 50/50 mix of O_2_ and N_2_O, along with 1 vol% sevoflurane inhalation. If the bispectral index score exceeded 60, midazolam (1 mg) was administered as a bolus. Upon completion of wound suturing, bilateral iliac artery balloons were removed, and muscle relaxation was reversed. Once spontaneous respiration and recovery of consciousness were confirmed, the patient was transferred to the recovery room. Patients were transferred from the recovery room to the ward only if their Modified Aldrete score was 8 or higher.

For patients undergoing cesarean section in the standard OR, all procedures were identical to those performed for the hybrid OR group, except that an awake 18-G peripheral intravenous line was placed instead of the right internal jugular central line. An intraoperative forced air warmer was applied based on the decision of the lead anesthesiologist.

### Variables and study outcome

Data obtained from electronic medical records were completely de-identified, ensuring the anonymity of all data. These records included patient baseline characteristics (age, body mass index, and gestational age at delivery); past medical history (including prior cesarean deliveries, hypertensive disorders [hypertension, gestational hypertension, preeclampsia, and eclampsia], diabetes mellitus [including gestational diabetes mellitus], thyroid disorders [hypothyroidism and hyperthyroidism]); and immediate preoperative temperature (tympanic membrane measured). Postoperative outcomes were also collected from the electronic medical records. These included intraoperative bleeding amount, intraoperative fluid administration (sum of crystalloid and colloid), operation duration (from intubation to end of skin closure), concurrent cesarean hysterectomy, total blood product transfusion (red blood cells, fresh frozen plasma, and platelet pack) during surgery, duration of postanesthetic care unit (PACU) stay, duration of hospital stay after surgery, perioperative complications (from surgery to hospital discharge, defined as intraoperative bladder injury, antibiotic use due to fever, and postoperative pleural effusion).

The primary objective of this study was to determine whether the type of OR (standard or hybrid) was associated with the incidence of immediate postoperative hypothermia, defined as a first tympanic membrane temperature below 36.0°C measured in the PACU. The secondary objective was to assess the effect of immediate postoperative hypothermia on the duration of PACU and postoperative hospital stays and the incidence of complications.

### Statistical analysis

Categorical variables are expressed as numbers (percentages) and were compared using the Pearson’s chi-squared test or Fisher’s exact test. Continuous variables with a non-normal distribution are presented as medians [interquartile ranges], and they were analyzed using the Wilcoxon rank-sum test. Continuous variables with a normal distribution are presented as mean (standard deviation), and were analyzed using the Welch’s t-test. The effect sizes are indicated by Cliff’s delta [17] for the postoperative outcomes presented as non-normally distributed continuous variables and relative risk for categorical variables.

For the primary outcome, we conducted univariate logistic regression analyses on variables known to be risk factors for postoperative hypothermia, including age, operation duration [18], body mass index [19], intraoperative fluid, intraoperative bleeding and transfusion [20], as well as surgeries performed in the Hybrid OR. Age and operation duration were categorized based on the median values of the entire dataset, while body mass index, intraoperative fluid, and intraoperative bleeding were categorized based on the 25th and 75th percentiles; intraoperative transfusion was categorized by the number of transfused packs. An overall p-value was calculated for variables with three categories, and variables with a p-value less than 0.25 were selected for adjusted analysis in a multivariable logistic regression model [21]. The final model was obtained through the backward stepwise elimination process using the ‘stats’ package in R. Before each variable elimination, the model prior to removal was compared with previous models using a partial likelihood ratio test, via the ‘lmtest’ package in R, to verify that the simplified model provided a fit comparable to that of the previous model; covariate interactions were checked in the final model. We evaluated the fit of the final model with the Hosmer–Lemeshow test and assessed its discrimination power using the receiver operating characteristic curve. An area under the receiver operating characteristic curve value greater than 0.8 was considered good; values of 0.60–0.80 were categorized as moderate, and values less than 0.60 were considered poor [22].

For the secondary outcomes, the durations of PACU and postoperative hospital stays of the immediate postoperative hypothermia and normothermia group within the Standard OR and Hybrid OR were compared using the Wilcoxon rank-sum test, excluding missing data. The incidence of complications was compared using the Fisher’s exact test. All statistical analyses were conducted using R Studio 4.2.2 (RStudio, Boston, MA, USA) and GraphPad Prism 10.0 (GraphPad Software, San Diego, CA, USA). Statistical significance was set at p < 0.05, except for the univariate logistic regression analysis (p <0.25).

## Results

### Study participants

**Fig 1:**
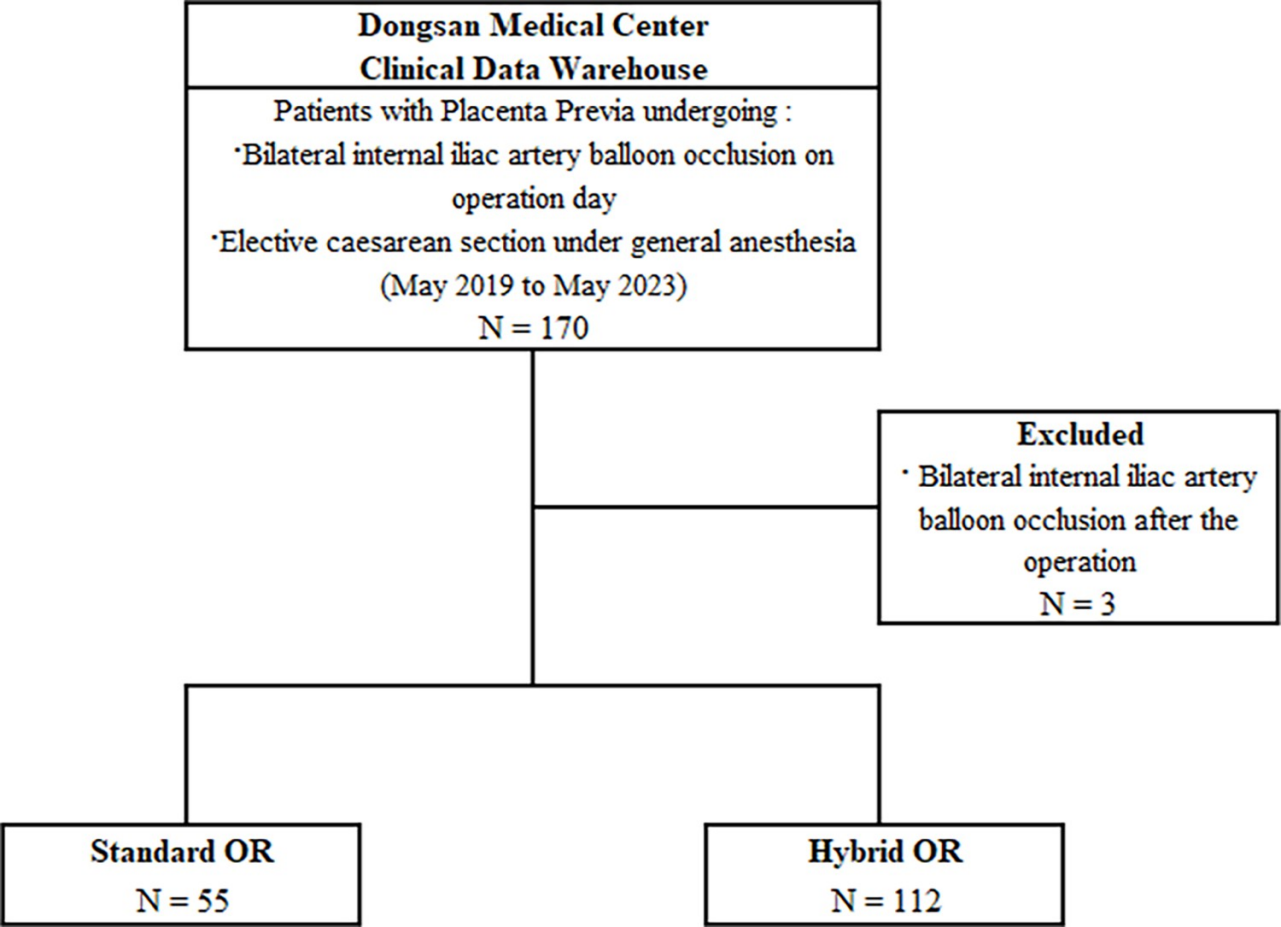
Study flow diagram.

Of the 170 patients with placenta previa who underwent elective cesarean section under general anesthesia between May 2019 and May 2023, three were excluded because bilateral internal iliac artery balloon occlusion was performed after the operation. Fifty-five patients underwent cesarean section in a standard OR, whereas 112 underwent the procedure in a hybrid OR (Fig 1). The baseline characteristics of the patients in the two groups were not clinically significantly different (Table 1).

**Table 1:** Baseline characteristics of the study population.

	Standard OR (n = 55)	Hybrid OR (n = 112)	p-value
**Age (years)**	34.6 (4.1)	35.2 (4.2)	0.408^a^
**Body mass index (kg/m** ^**2**^ **)**	26.3 (3.2)	26.8 (3.6)	0.353^a^
**Prior cesarean delivery (n)**			0.068^b^
** 0**	44 (80.0%)	78 (69.6%)	
** 1**	7 (12.8%)	30 (26.8%)	
** 2 or more**	4 (7.2%)	4 (3.6%)	
**Gestational age at delivery (weeks)**	36 [35–37]	36 [35–37]	0.505
**Gestational diabetes mellitus (n)**	8 (14.5%)	15 (13.4%)	0.816^b^
**Placenta accreta (n)**	34 (61.8%)	52 (46.4%)	0.088^b^
**Placenta increta (n)**	3 (5.5%)	7 (6.3%)	1.000^c^
**Placenta percreta (n)**	0 (0%)	1 (0.9%)	1.000^c^
**Hypertensive disorders (n)**	3 (5.5%)	1 (0.9%)	0.105^c^
**Diabetes mellitus (n)**	8 (14.5%)	15 (13.4%)	1.000^b^
**Thyroid disorder (n)**	4 (7.3%)	6 (5.4%)	0.731^c^
**Immediate preoperative temperature (°C)**	36.9 [36.8–37.0]	36.8 [36.7–36.9]	0.009*

Data are expressed as mean (standard deviation), median [interquartile range], and number (%). OR, operating room; hypertensive disorders include hypertension, gestational hypertension, preeclampsia, and eclampsia; thyroid disorders include hypothyroidism and hyperthyroidism; immediate preoperative temperature refers to tympanic membrane measured temperature

^a^ Welch’s t-test

^b^ Pearson’s chi-squared test

^c^ Fisher’s exact test

* Statistically significant.

### Postoperative outcome

The median postoperative temperature was lower for the hybrid OR group than in the standard OR group (36.3°C vs. 36.1°C, p = 0.028) with a small cliff’s delta effect size of -0.21. Immediate postoperative hypothermia (below 36.0°C) was also more prevalent in the hybrid than in the standard OR group (20% vs. 36.6%, p = 0.033), with a relative risk of 1.83 (95% confidence interval [CI] 1.02–3.28). Additionally, the PACU stay was longer for the hybrid OR group at an average of 30 min, compared with 25 minutes for the standard OR group (p = 0.002). The duration of surgery was longer for the hybrid OR group, at an average of 95 min, compared with 75 min in the standard OR group (p < 0.001). However, there were no significant differences between the groups related to intraoperative bleeding, volume of fluid administered, or number of intraoperative transfusion packs (Table 2).

**Table 2:** Postoperative outcome.

	Standard OR (n = 55)	Hybrid OR (n = 112)	p-value	Effect size ^a^	Relative risk (95% CI)
**Intraoperative bleeding (ml)**	900 [750–1175]	800 [700–1025]	0.082	-0.17	
**Intraoperative fluid (ml)**	1200 [1000–1500]	1400 [1100–1800]	0.054	0.18	
**Operation duration (min)**	75 [66–87]	95 [80–115]	< 0.001*	0.58	
**Cesarean hysterectomy (n)**	2 (3.6%)	2 (1.8%)	0.599^a^		0.49 (0.07,3.39)
**Intraoperative transfusion (pack)**					
** Red blood cell**	1 [0–2]	0 [0–1]	0.138	-0.13	
** Fresh frozen plasma**	0 [0–0]	0 [0–0]	0.061	-0.10	
** Platelet**	0 [0–0]	0 [0–0]	0.060	-0.03	
**Immediate postoperative temperature (°C)**	36.3 [36.0–36.4]	36.1 [35.8–36.3]	0.028*	-0.21	
**Immediate postoperative hypothermia (n)**	11 (20.0%)	41 (36.6%)	0.033^b^*		1.83 (1.02,3.28)
**Postanesthetic care unit stay (min)**	25 [22–30]	30 [25–38]	0.002*	0.31	
**Hospital stay after surgery (day)**	4 [4–4]	4 [4–4]	0.257	0.07	
**Perioperative complication (n)**	7 (12.7%)	11 (9.8%)	0.761^b^		0.77 (0.32,1.88)

Data are expressed as medians [interquartile ranges], numbers (%).

Immediate postoperative temperature, tympanic membrane temperature; perioperative complications such as duration from surgery to hospital discharge, intraoperative bladder injury, antibiotic use due to fever, and postoperative pleural effusion. ^a^ Cliff’s delta (< 0.33, small; 0.33–0.474, medium; > 0.474, large)

^a^ Fisher’s exact test

^b^ Pearson’s chi-squared test

* Statistically significant. OR, operating room; CI, confidence interval.

### Primary outcome

The results of the univariate logistic regression analysis are shown in Table 3. All seven variables were statistically significant (p < 0.25) and were included in the multivariable logistic regression analysis.

**Table 3:** Univariate logistic regression results of factors associated with postoperative hypothermia.

	N	Crude odd ratio	95% Confidence interval	p-value
**Hybrid OR**	55	2.31	1.08 to 4.96	0.032*
**Age (years)**	167			
** <35**	78	1.00	-	-
** ≥35**	89	1.83	0.93 to 3.59	0.078*
**Body mass index (kg/m** ^**2**^ **)**	167			0.228 ^a^ *
** <25**	68	1.00	-	-
** 25≤ mg/kg**^**2**^ **<30**	73	0.65	-0.06 to 1.36	0.235
** ≥30**	26	0.63	-0.36 to 1.63	0.369
**Intraoperative bleeding (ml)**	167			0.026 ^a^ *
** <700**	30	1.00	-	-
** 700≤ ml <1500**	119	1.15	0.25 to 1.52	0.528
** ≥1500**	18	2.75	2.04 to 3.98	0.032
**Intraoperative fluid (ml)**	167			0.009 ^a^ *
** <1100**	42	1.00	-	-
** 1100≤ ml <1800**	82	1.43	0.55 to 2.31	0.427
** ≥1800**	43	3.19	2.24 to 4.14	0.017
**Operation duration (min)**	167			
** <90**	81	1.00	-	-
** ≥90**	86	1.81	0.93 to 3.53	0.082*
**Intraoperative transfusion (pack)**	167	1.23	1.06 to 1.43	0.007*

^a^ Overall p-value for categorical analysis

* Statistically significant. N, number

Multivariable backward stepwise logistic regression analysis incorporating group, age, surgical duration, body mass index, amount of intraoperative bleeding, amount of intraoperative fluid, and intraoperative blood transfusion packs was performed (see 1 in S1 Text). We compared the models before and after each variable removal using the partial likelihood ratio test, and found no significant difference (p > 0.05, see 2 in S1 Text). Consequently, the final model, which included four variables—group, intraoperative transfusion, operation duration, and body mass index—was selected for its parsimony. No interactions were observed between the variables in the final model (see 3 in S1 Text).

**Fig 2:**
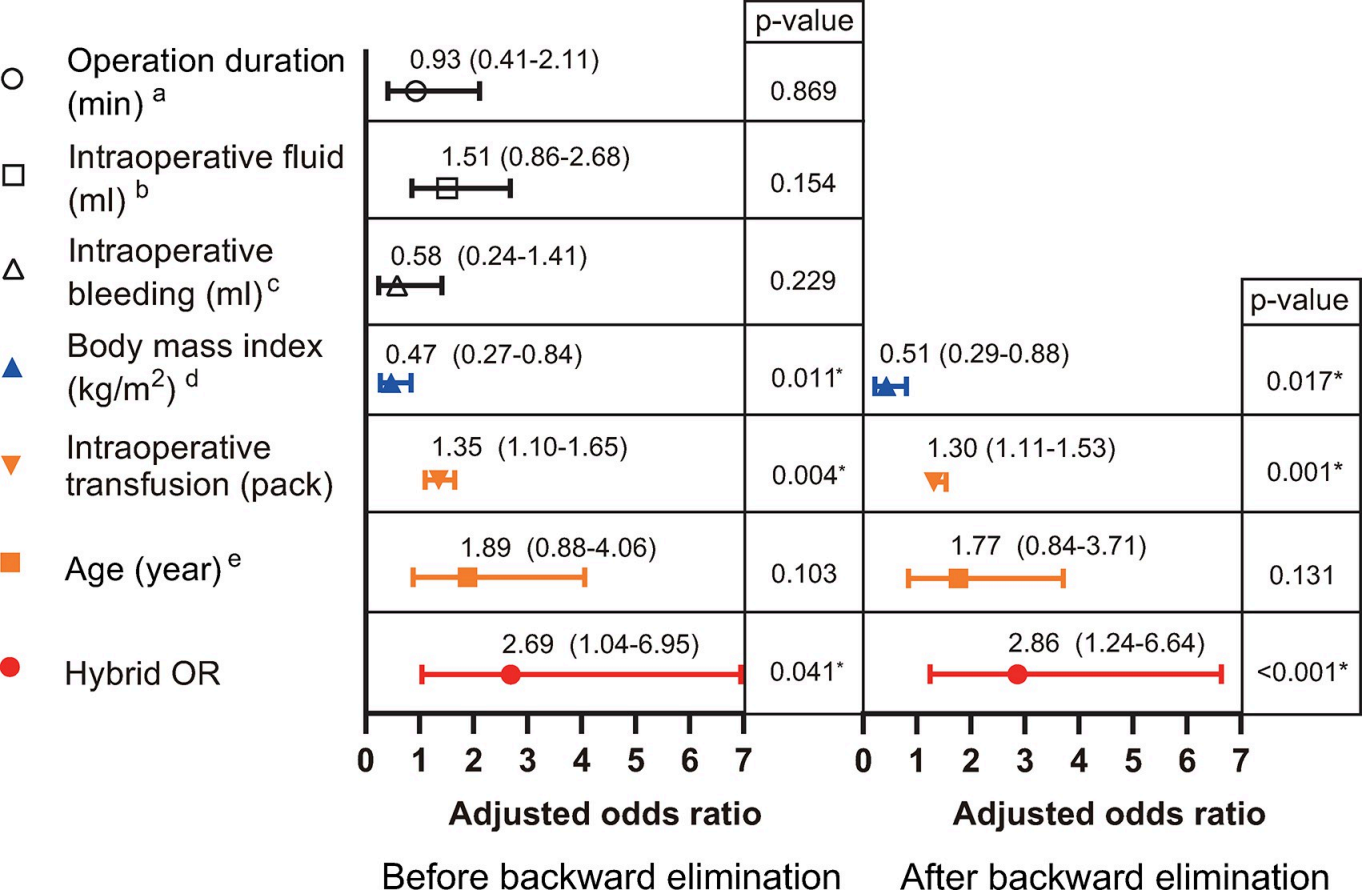
Multivariable logistic regression results after adjusting factors associated with postoperative hypothermia. ^a^ Adjusted for categorical factors (<90; ≥90). ^b^ Adjusted for categorical factors (<1100; 1100 ≤ ml < 1800; ≥1800). ^c^ Adjusted for categorical factors (<700; 700 ≤ ml < 1500; ≥1500). ^d^ Adjusted for categorical factors (<25; 25 ≤ mg/kg^2^ < 30; ≥30). ^e^ Adjusted for categorical factors (<35; ≥35). * Statistically significant.

In the multivariable analysis, after backward elimination, patient undergoing surgery in the hybrid OR was significantly associated with an increased risk of immediate postoperative hypothermia (adjusted odds ratio 2.86; 95% CI 1.24–6.64, p < 0.001). Likewise, the number of intraoperative transfusion packs was significantly associated with immediate postoperative hypothermia (adjusted odds ratio 1.30; 95% CI 1.11–1.53, p = 0.001). However, age was not associated with immediate postoperative hypothermia (adjusted odds ratio, 1.77; 95% CI 0.84–3.71, p = 0.131). Moreover, a higher body mass index offered a protective effect against immediate postoperative hypothermia (adjusted odds ratio, 0.51; 95% CI 0.29–0.88, p = 0.017) (Fig 2).

**Fig 3:**
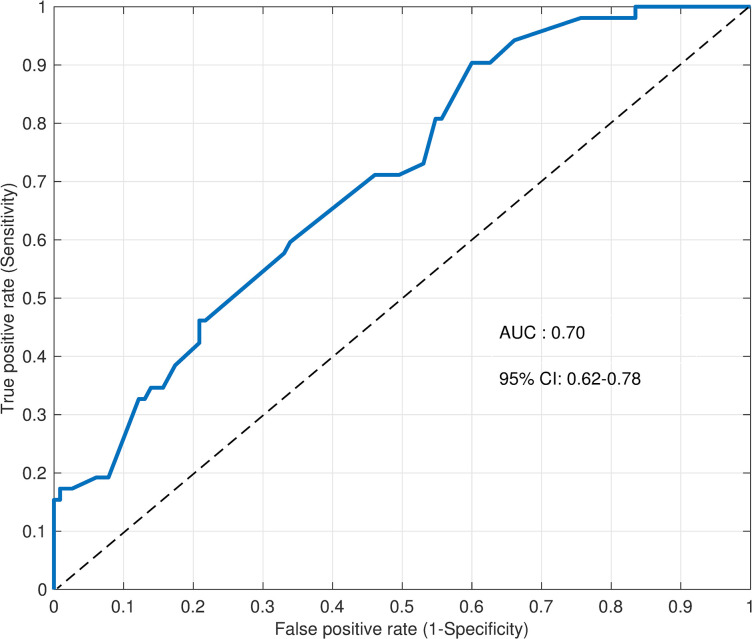
Receiver operating characteristic curve for the final model. AUC, area under the receiver operating characteristic curve; CI, confidence interval. AUC > 0.8, good predictive accuracy; 0.6 ≤ AUC ≤ 0.8, moderate accuracy; AUC < 0.6, poor accuracy.

The Hosmer–Lemeshow test for the final model indicated a good fit (p = 0.088, see 4 in S1 Text), and the final model’s area under the receiver operating characteristic curve was 0.70 (95% CI 0.62–0.78), showing moderate discriminatory ability (Fig 3).

### Secondary outcomes

In the Standard OR group (n = 55), 44 patients experienced immediate postoperative hypothermia, while 11 exhibited normothermia. In the Hybrid OR group (n = 112), the numbers were 71 and 41, respectively. Six patients in the Standard OR group had missing PACU stay data—three each in the normothermia and hypothermia groups. Additionally, one patient in the Hybrid OR group had missing PACU stay data due to a direct transfer to the ward. No data were missing for the hospital stay after surgery.

Patients with immediate postoperative hypothermia in the Standard OR group (n = 8) had a longer median PACU stay of 30 (range 23–48) min, compared with the 25 (range 20–65) min for the normothermia (n = 41) group (p = 0.045). In the Hybrid OR group, patients with immediate postoperative hypothermia (n = 41) had a longer median PACU stay of 40 (range 25–90) minutes, compared to the 26 (range 20–65) minutes for those with normothermia (n = 70), a difference that was statistically significant (p < 0.001). In the Standard OR group, patients with hypothermia (n = 11) had a median hospital stay after surgery of 4 days (range 3–16), compared to the median of 4 days (range 3–5) for those with normothermia (n = 44), showing a statistically significant difference (p = 0.021). Similarly, in the Hybrid OR group, the median hospital stay after surgery for patients with hypothermia (n = 41) was 4 days (range 4–11), versus 4 days (range 3–5) for those with hypothermia (n = 71), also with a statistically significant difference (p = 0.019) (Table 4).

**Table 4:** Secondary outcomes of the immediate postoperative normothermia and hypothermia within groups.

	Standard OR (n = 49)			Hybrid OR (n = 111)		
	**Normothermia (n = 41)**	**Hypothermia (n = 8)**	**p-value**	**Normothermia (n = 70)**	**Hypothermia (n = 41)**	**p-value**
**PACU stay**	25 (20–65)	30 (23–48)	0.045*	26 (20–65)	40 (25–90)	<0.001*
	**Standard OR (n = 55)**			**Hybrid OR (n = 112)**		
	**Normothermia (n = 44)**	**Hypothermia (n = 11)**	**p-value**	**Normothermia (n = 71)**	**Hypothermia (n = 41)**	**p-value**
**Hospital stay after surgery**	4 (3–5)	4 (3–16)	0.019*	4 (3–5)	4 (4–11)	0.021*

Data are expressed as median (range). PACU = postanesthetic care unit; Hypothermia = immediate postoperative hypothermia; Normothermia = immediate postoperative normothermia

* Statistically significant

**Fig 4:**
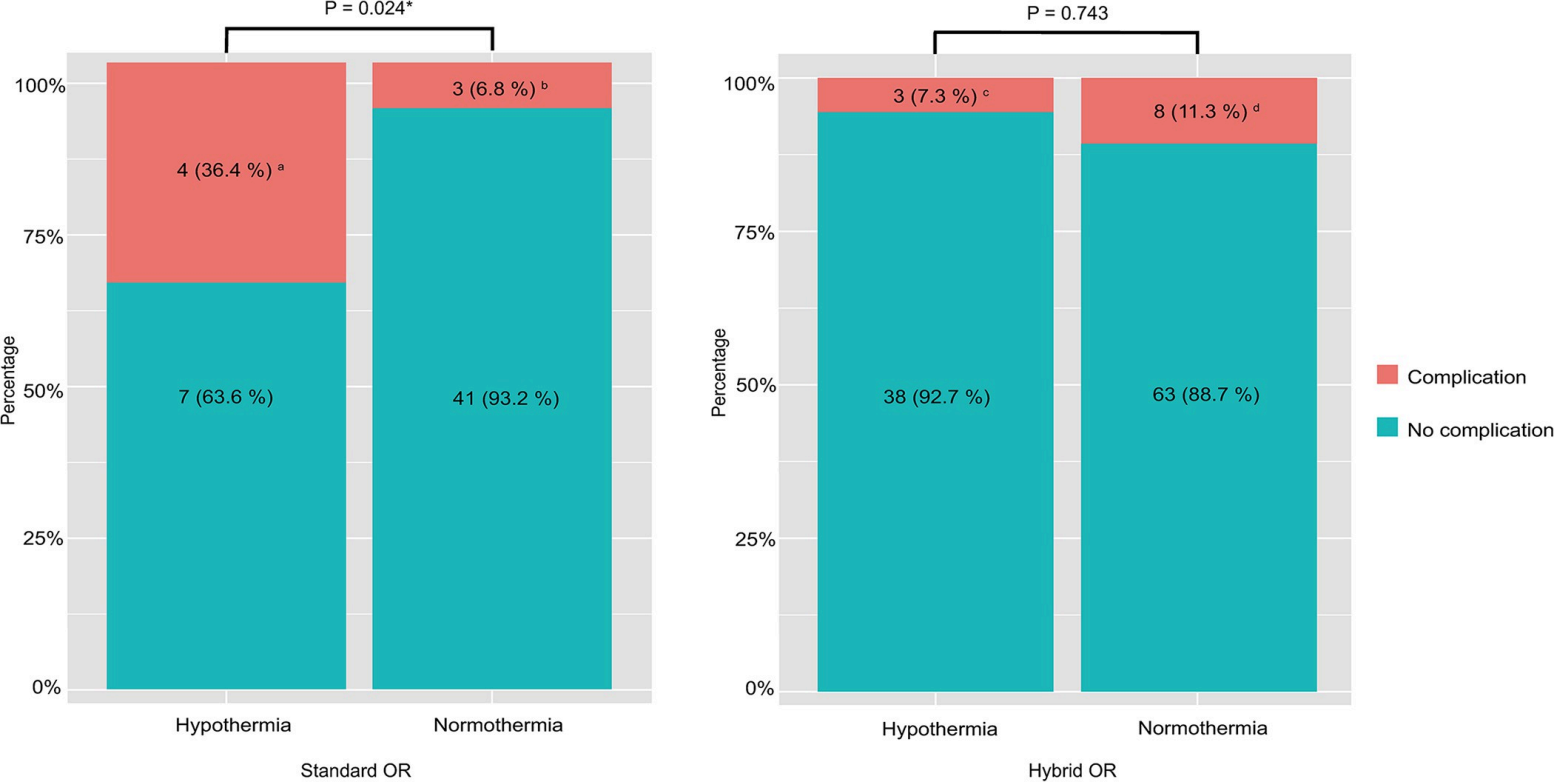
Complication rates of the postoperative immediate hypothermia and normothermia within groups. Data are expressed as number (percentage). ^**a**^ Two individuals had pleural effusion, one had intraoperative bladder injury, and one had intraoperative bladder injury and fever due to postoperative urinary tract infection. ^**b**^ Three individuals had pleural effusion. ^c^ Two individuals had pleural effusion and one had intraoperative bladder injury. ^d^ Six individuals had pleural effusion, one had intraoperative bladder injury and pleural effusion, and one had fever due to bacteremia. Hypothermia = immediate postoperative hypothermia; Normothermia = immediate postoperative normothermia. * Statistically significant.

The incidence of complications was significantly different between hypothermia and normothermia in the Standard OR group (36.4% vs 6.8%, p = 0.024). However, the incidence of complications was not significantly different between normothermia and hypothermia in the Hybrid OR group (7.3% vs 11.3%, p = 0.743) (Fig 4).

## Discussion

In this retrospective study, we observed that the type of OR (standard vs. hybrid) and volume of intraoperative transfusion were significantly associated with immediate postoperative hypothermia. Interestingly, a higher body mass index was found to have a protective effect against postoperative hypothermia. Furthermore, patients with immediate postoperative hypothermia had a prolonged stay in the PACU and an extended postoperative hospital stay. However, no association was observed between immediate postoperative hypothermia and the incidence of complications in the Hybrid OR group.

To the best of our knowledge, this is the first study to demonstrate that pregnant women with placenta previa who undergo elective cesarean section in a hybrid OR under general anesthesia are at a higher risk of immediate postoperative hypothermia than those in a standard OR. In our study, the standard and hybrid ORs maintained identical temperature and humidity settings (25°C, 40–60%). However, surgery performed in the hybrid OR was associated with immediate postoperative hypothermia (adjusted odds ratio 2.86, p < 0.001). This is consistent with previous findings, in which surgeries in hybrid ORs for pediatric cases were linked to increased hypothermia [9, 10]. However, the average temperature and humidity in these studies on hybrid ORs were low (18°C, 40%). A possible explanation for the association between the hybrid OR and immediate postoperative hypothermia in our study may be the difference in room size. Hybrid ORs are typically larger than standard ORs, often because of the need for additional equipment such as a C-arm. The room size of the hybrid OR in our study was 87 m^2^, which was significantly larger than the 35 m^2^ standard OR. Consequently, a hybrid OR requires more heat to alter air temperature [23]. Therefore, the actual ambient temperature in the hybrid OR is lower than that in the standard OR, leading to greater radiation heat loss [24].

In our study, a longer duration of surgery was not associated with immediate postoperative hypothermia (adjusted odds ratio 0.93, p = 0.869). Some previous studies have reported the duration of surgery as a potential risk factor for postoperative hypothermia, but these studies involved patients from various surgical departments [25, 26]. The durations of surgery reported in these studies were also notably longer, with ranges of 150–420 min [25] and 120–240 min [26] compared with the 50–220 min duration observed in our study (S1 Fig). Notably, our findings are consistent with those of a previous retrospective study on gynecologic patients, in which the durations of surgery were between 65 and 160 min [19]. That study also concluded that the duration of surgery was not a significant risk factor for postoperative hypothermia [19].

Interestingly, following multivariate backward stepwise elimination, age was not associated with immediate postoperative hypothermia (adjusted odds ratio 1.77, p = 0.131). Despite age being a well-known risk factor for postoperative hypothermia, previous literature suggests a threshold effect, with increased risk typically observed in individuals over 50 years old [26, 27]. In our study, all patients were under 50, which may explain why age was not a risk factor, and our findings were consistent with an another study that also reported no association between age and postoperative hypothermia [19]; notably, this cited study also included middle-aged women.

Some studies have suggested that a high body mass index has a protective effect against postoperative hypothermia [18, 28]. Consistent with these findings, our study revealed that a higher body mass index was associated with a decreased risk of immediate postoperative hypothermia (adjusted odds ratio, 0.90, p = 0.023).

The consequences of immediate postoperative hypothermia are consistent with the findings of previous studies. Intraoperative hypothermia has been shown to prolong PACU and hospital stays [11–13]. Although one prospective study found an association between hypothermia and postoperative complications [25], our study did not find such a correlation in the Hybrid OR group. This discrepancy may be because of the differences in the definition of hypothermia (below 35.0°C vs. below 36.0°C). Temperatures below 35.0°C have been associated with complications, according to a previous study [25]. However, in our study, no patients experienced a postoperative temperature lower than 35.0°C. Furthermore, a meta-analysis by Xu et al. [29] indicated that intraoperative hypothermia, defined as 36.0°C, was not associated with significant clinical outcomes, with the exception of shivering occurrence. Although the Standard OR group did exhibit a significant association between immediate postoperative hypothermia and complications, this finding does not align with previous studies and may have been influenced by the large discrepancy in sample sizes within the group (11 vs 44); thus, its clinical significance may be questionable.

This study had some limitations. First, because the decision to warm the patient was made by the individual anesthesiologist in charge of the room, bias may have been introduced. Therefore, future prospective randomized studies with controlled warming protocols are required to ascertain the effects of hybrid OR on postoperative hypothermia. Second, there was a large sample size difference between the hypothermia and normothermia groups within the Standard OR group. This discrepancy may have led to statistically significant results that are not clinically significant [30]. Lastly, this study was conducted retrospectively at a single center. A larger prospective multicenter study is required to generalize our findings.

In conclusion, although the duration of surgery in the hybrid OR was longer than that in the standard OR, it did not significantly affect the risk of immediate postoperative hypothermia. Our study suggests that the intrinsic characteristics of the hybrid OR may pose a risk for postoperative hypothermia. Given the association between hypothermia and extended PACU stay and hospitalization, preventing hypothermia in patients undergoing surgery in a hybrid OR is crucial.

## Supporting information

S1 Text Backward stepwise logistic regression, partial likelihood ratio tests, and Hosmer–Lemeshow fit assessment. (DOCX)

S1 Fig Histogram of durations of surgery for the study participants. (TIF)

S1 Dataset (XLSX)
