# Supplementary material for: Hybrid operating rooms and the risk of postoperative hypothermia in pregnant women with placenta previa: A retrospective cohort study
Source: PLoS One. 2024 Jun 25;19(6):e0305951. doi: 10.1371/journal.pone.0305951 (PMC11198747; doi:10.1371/journal.pone.0305951)
Supplement: S1 Text — (DOCX) [file pone.0305951.s001.docx]

1. R script for backward stepwise logistic regression result incorporating seven variables (group, age, surgical duration, body mass index, amount of intraoperative bleeding, amount of intraoperative fluid, and intraoperative blood transfusion packs)

> step(multivariate_logic,direction='backward')

Start: AIC=197.78

post_hypothermia ~ age_scaled + group + op_dur_90 + bmi_scaled +

bleeding_scaled + fluid_scaled + intraop_trans

Df Deviance AIC

- op_dur_90 1 181.81 195.81

- bleeding_scaled 1 183.22 197.22

<none> 181.78 197.78

- fluid_scaled 1 183.85 197.85

- age_scaled 1 184.50 198.50

- group 1 186.24 200.24

- bmi_scaled 1 188.94 202.94

- intraop_trans 1 191.17 205.17

Step: AIC=195.81

post_hypothermia ~ age_scaled + group + bmi_scaled + bleeding_scaled +

fluid_scaled + intraop_trans

Df Deviance AIC

- bleeding_scaled 1 183.31 195.31

<none> 181.81 195.81

- fluid_scaled 1 183.87 195.87

- age_scaled 1 184.50 196.50

- group 1 186.99 198.99

- bmi_scaled 1 188.94 200.94

- intraop_trans 1 191.23 203.23

Step: AIC=195.31

post_hypothermia ~ age_scaled + group + bmi_scaled + fluid_scaled +

intraop_trans

Df Deviance AIC

- fluid_scaled 1 184.57 194.57

<none> 183.31 195.31

- age_scaled 1 185.49 195.49

- group 1 188.80 198.80

- bmi_scaled 1 189.81 199.81

- intraop_trans 1 191.48 201.48

Step: AIC=194.57

post_hypothermia ~ age_scaled + group + bmi_scaled + intraop_trans

Df Deviance AIC

<none> 184.57 194.57

- age_scaled 1 186.88 194.88

- bmi_scaled 1 190.79 198.79

- group 1 191.24 199.24

- intraop_trans 1 197.04 205.04

Final model include four variables (age, group, body mass index and intraoperative transfusion packs)

2. R script for partial likelihood ratio test. Models before and after variable removal was compared.

> library(lmtest)

> lrtest(multivariate_logic,multivariate_logic_step1)

Likelihood ratio test

Model 1: post_hypothermia ~ age_scaled + group + op_dur_90 + bmi_scaled +

bleeding_scaled + fluid_scaled + intraop_trans

Model 2: post_hypothermia ~ age_scaled + group + bmi_scaled + bleeding_scaled +

fluid_scaled + intraop_trans

#Df LogLik Df Chisq Pr(>Chisq)

1 8 -90.890

2 7 -90.904 -1 0.0272 0.8689

> lrtest(multivariate_logic_step1,multivariate_logic_step2)

Likelihood ratio test

Model 1: post_hypothermia ~ age_scaled + group + bmi_scaled + bleeding_scaled +

fluid_scaled + intraop_trans

Model 2: post_hypothermia ~ age_scaled + group + bmi_scaled + fluid_scaled +

intraop_trans

#Df LogLik Df Chisq Pr(>Chisq)

1 7 -90.904

2 6 -91.657 -1 1.5049 0.2199

> lrtest(multivariate_logic_step2,multivariate_logic_step3)

Likelihood ratio test

Model 1: post_hypothermia ~ age_scaled + group + bmi_scaled + fluid_scaled +

intraop_trans

Model 2: post_hypothermia ~ age_scaled + group + bmi_scaled + intraop_trans

#Df LogLik Df Chisq Pr(>Chisq)

1 6 -91.657

2 5 -92.287 -1 1.2611 0.2615

In each step, Model 2 (after variable removal) performs as well as Model 1 (before variable removal, p > 0.05)

3. R script for checking interactions among covariates

(1) interaction between ‘group’ and ‘intraoperative blood transfusion packs’

> model.interaction_1 <- glm(post_hypothermia~age_scaled + group + bmi_scaled + intraop_trans+

intraop_trans:group, family=binomial, data=Data_R1, na.action=na.exclude)

> lrtest(backward.multivariate_logic,model.interaction_1)

Likelihood ratio test

Model 1: post_hypothermia ~ age_scaled + group + bmi_scaled + intraop_trans

Model 2: post_hypothermia ~ age_scaled + group + bmi_scaled + intraop_trans +

intraop_trans:group

#Df LogLik Df Chisq Pr(>Chisq)

1 5 -92.287

2 6 -91.877 1 0.8194 0.3653

(2) Interaction between ‘intraoperative blood transfusion packs’ and ‘age’

> model.interaction_2 <- glm(post_hypothermia~age_scaled + group + bmi_scaled + intraop_trans+ intraop_trans:age_scaled, family=binomial, data=Data_R1, na.action=na.exclude)

> lrtest(backward.multivariate_logic,model.interaction_2)

Likelihood ratio test

Model 1: post_hypothermia ~ age_scaled + group + bmi_scaled + intraop_trans

Model 2: post_hypothermia ~ age_scaled + group + bmi_scaled + intraop_trans +

intraop_trans:age_scaled

#Df LogLik Df Chisq Pr(>Chisq)

1 5 -92.287

2 6 -92.198 1 0.1785 0.6727

(3) Interaction between ‘body mass index’ and ‘age’

> model.interaction_3 <- glm(post_hypothermia~age_scaled + group + bmi_scaled + intraop_trans+ bmi:age_scaled, family=binomial, data=Data_R1, na.action=na.exclude)

> lrtest(backward.multivariate_logic,model.interaction_3)

Likelihood ratio test

Model 1: post_hypothermia ~ age_scaled + group + bmi_scaled + intraop_trans

Model 2: post_hypothermia ~ age_scaled + group + bmi_scaled + intraop_trans +

bmi:age_scaled

#Df LogLik Df Chisq Pr(>Chisq)

1 5 -92.287

2 6 -91.513 1 1.5474 0.2135

When comparing the model that includes an interaction term with the final model, no significant difference was observed (p > 0.05)

4.Hosmer-Lemeshow test result for final model

>library(ResourceSelection)

>y <- Data_R1$post_hypothermia

>hoslem.test(multivariate_logic_step3$y,fitted(backward.multivariate_logic_step3))

Hosmer and Lemeshow goodness of fit (GOF) test

data: multivariate_logic_step3$y, fitted(multivariate_logic_step3)

X-squared = 12.402, df = 7, p-value = 0.0881

A p-value greater than 0.05 suggests that there is no significant difference between the observed and predicted values.
