# Supplementary figures and images for: Hybrid operating rooms and the risk of postoperative hypothermia in pregnant women with placenta previa: A retrospective cohort study
Source: PLoS One. 2024 Jun 25;19(6):e0305951. doi: 10.1371/journal.pone.0305951 (PMC11198747; doi:10.1371/journal.pone.0305951)

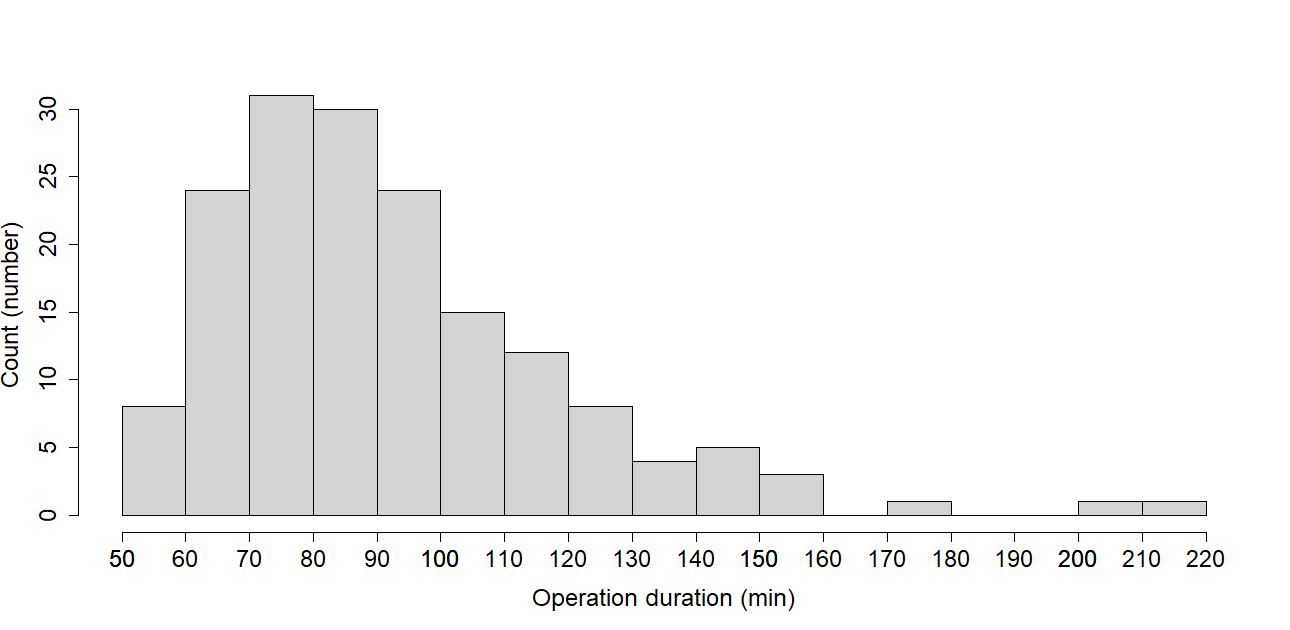

Supplement: S1 Fig — (TIF) [file pone.0305951.s002.tif]
